# Psychometric properties of the 12-item Stroke-Specific Quality of Life Scale among stroke survivors in Hong Kong

**DOI:** 10.1038/s41598-023-28636-7

**Published:** 2023-01-27

**Authors:** Ted C. T. Fong, Temmy L. T. Lo, Rainbow T. H. Ho

**Affiliations:** 1 Centre on Behavioral Health, University of Hong Kong, 2/F, The Hong Kong Jockey Club Building for Interdisciplinary Research, 5 Sassoon Road, Pokfulam, Hong Kong, China; 2 Department of Social Work and Social Administration, University of Hong Kong, Hong Kong, China

**Keywords:** Psychology, Health care, Risk factors, Signs and symptoms

## Abstract

The present study examined the psychometric properties of the 12-item Stroke-Specific Quality of Life Scale (SSQOL-12) in 184 stroke survivors in Hong Kong. The participants completed a self-report questionnaire including the SSQOL-12, SF-12 health survey, and validating variables at baseline and 148 stroke survivors completed SSQOL-12 two months later. Confirmatory factor analysis investigated the construct validity, reliability, and measurement invariance of SSQOL-12 across two months. Concurrent, convergent, and divergent validity was examined with respect to SF-12, hope, self-esteem, functional disability, anxiety, and depression. The original 2-factor model did not reveal a superior fit over the 1-factor model and a modified 1-factor model provided an acceptable fit to the data in both waves. The SSQOL-12 factor displayed substantial factor loadings (λ = 0.40–0.87), good internal consistency (Ω = 0.88), temporal stability (r = 0.70), and scalar measurement invariance across time. Stroke-specific quality of life was significantly associated with higher levels of SF-12, hope, and self-esteem and lower levels of functional disability, anxiety, and depression. Most correlations remained significant after controlling for demographic covariates and SF-12. The present findings offered empirical support for the validity and reliability of the unidimensional structure for SSQOL-12 as a measure of stroke-specific quality of life among stroke survivors.

## Introduction

Stroke is a neurocardiovascular disease with two major types of ischemic and haemorrhagic stroke^1^. It is a universal public health issue^2^ and carries significant economic burden to the society in terms of post-stroke care for the survivors^3^. Traditionally, stroke survivors have been the older adults who were substantially impaired and were in need of continuous rehabilitation treatments. The past two decades have witnessed a changing landscape of stroke in that stroke survivors are becoming younger, more physically active, and suffer from neurologically milder impairments^4^. Although these younger stroke survivors likely suffer less severe neurological impairments in domains of quality of life such as motor, speech, and mobility, the occurrence of neuropsychiatric symptoms is common among them^5^. Around one-third of stroke survivors suffer from persistent deficits in engagement, autonomy, and fulfilling societal roles^6^.

The younger stroke survivors often have a chronic stroke recovery process from regaining independent living to returning to work and establishing self-management^7^. Their health-related quality of life is likely adversely impacted by the associated stroke deficits during the recovery process^8^ and returning to pre-stroke functional and work status involves substantial changes to roles and social relationships^9^. The associated psychosocial risk factors, such as fear and uncertainty, could predispose them to emotional distress^10,11^. A review study^12^ found psychosocial factors to be associated with increased risks of stroke and transient ischemic attack in populations of all ages. It is therefore essential to have a valid and reliable stroke-specific tool for a comprehensive assessment of the quality of life in physical and psychosocial domains.

The 49-item Stroke-Specific Quality of Life (SSQOL) Scale was developed by Williams et al.^13^ and has been validated in various samples of stroke survivors^14,15^. Since the administration of the 49-item scale is cumbersome for stroke survivors in clinical settings, a 12-item brief version (SSQOL-12) was developed by selecting the item with the strongest item-total correlation from each of the 12 domains^16^. Compared to the common Short Form 12-item (SF-12) Health Survey^17^, the SSQOL-12 includes a holistic assessment of the functioning of stroke survivors in domains such as cognition, mobility, language, and vision that are prognostic factors of successful stroke rehabilitation^18–20^. The Chinese version of the SSQOL-12 was derived from 186 haemorrhagic stroke survivors in Hong Kong^21^. Despite the satisfactory internal consistency, criterion validity, and convergent validity for the SSQOL-12^22–24^, there was less support for its construct validity.

First, the scale development studies^16,21^ did not empirically test the factor structure. Although subsequent studies^14,15^ suggested a two-factor (physical and psychosocial) structure on the SSQOL-12, these studies are subject to methodological issues such as using principal component analysis and orthogonal varimax rotation^25^. These obsolete analytic methods could lead to an incorrect number of factors with distorted factor structures^26,27^. Second, a psychometric study^28^ obtained a mediocre fit for the 2-factor model for Wong’s SSQOL-12 version. As shown in Table 1, 9 out of the 12 items displayed ceiling effects with at least one-fourth of the respondents endorsing the highest response. Despite the considerable degree of deviation from the normality assumption, the authors treated the items as skewed continuous variables using the robust maximum likelihood estimator. This practice did not account for the items’ asymmetrical distributions and could yield incorrect results^29^. The factor structure of the SSQOL-12 should be examined using categorical methods on ordinal items.

**Table 1:** Demographic, clinical, and psychological profiles of the study sample at baseline.

Categorical variables	N (%)		
Gender			
Male/female	113 (61)/71 (39)		
Marital status			
Married/single	109 (60)/74 (40)		
Education level			
Primary/Secondary/Tertiary	26 (14)/110 (60)/47 (26)		
Stroke type			
Hemorrhagic/Ischemic	76 (43)/102 (57)		
Modified rankin scale			
0 or 1/2/3 or 4	37 (20)/94 (51)/53 (29)		

Continuous variables	Range	Skewness	Mean (SD)
Age (years)	23–64	− 1.12	55.2 (7.92)
Onset time of stroke (years)	0–25	2.13	3.83 (4.25)
Anxiety	0–20	0.52	6.73 (4.13)
Depression	0–20	0.42	7.28 (3.89)
Hope	6–48	− 0.85	33.5 (9.08)
Self-esteem	10–39	− 0.09	26.5 (5.20)
SF-12	24.9–58.0	0.05	41.6 (7.13)
Stroke-specific QoL	17–60	0.10	39.4 (9.60)

N = 184; SF-12 = 12-item Short Form Health Survey; QoL = quality of life.

Third, the chronic nature of the stroke recovery process often involves the investigation of the temporal change in SSQOL of the survivors. Longitudinal measurement invariance refers to the stability of measurement parameters (factor loadings and item thresholds) over time and is an important psychometric property of a measurement scale^30^. Temporal changes in the scale scores can be attributed to changes in the underlying construct only if longitudinal measurement invariance holds^31^ and longitudinal non-invariance would bias inferences on the temporal changes in the SSQOL. The SSQOL-12 should show an invariant measurement structure to ensure unbiased comparisons of the latent SSQOL scores across time. However, the recovery trajectory of stroke^32^ could change the survivors’ subjective perceptions toward oneself and the body and potentially alter the measurement parameters of the SSQOL-12. To our knowledge, no existing studies have investigated the longitudinal measurement invariance of the SSQOL-12. There is currently a lack of systematic studies to evaluate the psychometric properties of the SSQOL-12 in the Chinese context. Such a systematic evaluation is vital for an accurate assessment of the quality of life in stroke-specific domains.

In view of the research gaps, the present study aimed to evaluate the psychometric properties of the SSQOL-12 in Chinese stroke survivors. The first objective of the study was to evaluate the construct validity and reliability of the SSQOL-12. The second objective was to investigate the measurement invariance of the SSQOL-12 factors across time. The third objective aimed to examine the concurrent, convergent, divergent validity of the SSQOL-12 with regard to the SF-12, coping (hope and self-esteem), and opposing constructs (degree of disability, anxiety, and depression), respectively. For concurrent validity, we hypothesized that SSQOL would be positively and moderately to strongly correlated with SF-12 as a generic quality of life measure. Patients with better quality of life have shown better emotional functioning and coping^33,34^. For convergent validity, we hypothesized that SSQOL would be positively and moderately correlated with hope and self-esteem. For divergent validity, we hypothesized that SSQOL would be negatively and moderately correlated with the degree of disability, anxiety, and depression.

## Methods

### Sample

The present study sample comprised 184 stroke survivors in Hong Kong, who were recruited under convenience sampling in centers of the community rehabilitation network and a local hospital from 2018 to 2021. The present study had a larger sample size than those reported in previous psychometric studies (N = 114–146)^35–37^ and a subject to item ratio of 184:12 = 15.3. The inclusion criteria were experience of a stroke event, age between 18 and 64, and ability to understand Chinese. The exclusion criteria were the presence of psychiatric disorders that required hospitalization, diagnosis of no disability (modified Rankin Scale [mRS] score = 0) or severe disability (mRS score = 5). Participation was completely voluntary and all participants provided written informed consent. The participants received a HKD 50 (USD 6.5) voucher incentive upon completion of the assessment in each wave. Ethical approval was obtained from the Human Research Ethics Committee of the University of Hong Kong (EA1702058) and the Institutional Review Board of the University of Hong Kong/Hospital Authority Hong Kong West Cluster (UW18-467) and East Cluster (HKECREC-2019-111). The present study was reported with reference to the COSMIN study design checklist for patient-reported outcome measurement instruments^38^ on the construct validity, convergent validity, internal consistency, and measurement invariance of the SSQOL-12.

### Procedures and measures

The participants completed a self-report questionnaire on the SSQOL-12, SF-12, and mental health variables at baseline (Time 0). The participants took approximately 15 min to complete the questionnaire. A total of 148 participants completed the SSQOL-12 2 months later (Time 1). The short form of Stroke-specific Quality of Life (SSQOL-12) is a measure on the disease-specific quality of life in stroke survivors^21^. The 12 items inquired about the extent of interference in quality of life (QoL) as a result of stroke and were rated on a 5-point Likert format from 1 = “agree completely” to 5 = “disagree completely”. The scale yielded two subscales in physical (7 items) and psychosocial domains (5 items). The modified Rankin Scale (mRS) measured the degree of disability in the daily activities of the respondents who suffered from stroke^39^. The present study adopted the simplified mRS Questionnaire to assess the level of disability with the degree of disability rated on a 6-point format from 0 = “no symptoms/disability” to 5 = “severe disability”.

The SF-12 Health Survey^17^ was used to assess the generic QoL of the participants. Out of a theoretical range from 0 to 100, the mean scores for physical QoL and mental QoL ranged from 39.2 to 46.3 and 45.8 to 54.9, respectively, in previous studies among stroke survivors^40,41^. The physical and mental QoL scores were averaged to produce the total SF-12 score. Anxiety and depressive symptoms were evaluated by the 14-item Hospital Anxiety and Depression Scale^42^. The items were answered in a 4-point Likert format and the two subscales had a theoretical range from 0 to 21. The 6-item State Hope Scale^43^ measured the respondents’ level of hope in terms of agency and pathways. The items were rated on an 8-point Likert format from 1 = “Definitely false” to 8 = “Definitely true”. The total hope score had a theoretical range from 8 to 48. Rosenberg Self-Esteem Scale^44^ was used to assess the respondents’ level of self-esteem. The 10 items were rated on a 4-point Likert format from 1 = “Disagree very much” to 4 = “Agree very much”. The total self-esteem score had a theoretical range from 10 to 40. The present sample showed satisfactory to good levels of internal consistency (Cronbach’s α = 0.74–0.91) for the measures on SF-12, anxiety, depression, hope, and self-esteem.

### Ethical approval

All procedures contributing to this work complied with the ethical standards of the relevant national and institutional committees on human experimentation and with the Helsinki Declaration of 1975, as revised in 2008. Ethics approval was obtained from the Human Research Ethics Committee of the University of Hong Kong (Reference number: EA1702058). The objectives, data collection procedure, and issues regarding confidentiality and anonymity were explained to the participants. Informed consent was obtained from all of the study participants.

### Data analysis

Four of the 12 SSQOL-12 items showed substantial ceiling effects with at least 25% of the respondents endorsing the maximum level. The robust weighted least square (WLSMV) estimator is preferred over the maximum likelihood estimator for modeling items with asymmetric category thresholds to avoid biased factor loadings and standard errors^29^. The present study modeled the SSQOL items as ordinal categorical variables using the WLSMV estimator. The psychometric properties of the SSQOL-12 were examined in three steps.

First, the 1-factor and 2-factor structure of the SSQOL-12 were examined in Time 0 via confirmatory factor analysis (CFA) in Mplus 8.5^45^. Problematic items with no substantial factor loadings (λ < 0.40) were iteratively removed from the model^46^. Model fit was appraised using the following cutoff criteria^47^ on the fit indices: comparative fit index (CFI) and Tucker-Lewis index (TLI) ≥ 0.95, root mean square error of approximation (RMSEA) and standardized root mean square residuals (SRMR) ≤ 0.08. In case of inadequate model fit, modification indices were inspected together with the expected parameter changes. Model modifications that had theoretical justifications would be added to the CFA model. Model fit of nested models was compared using the chi-square difference test. The optimal factor structure derived in Time 0 would be tested in Time 1.

Internal consistency of the SSQOL-12 factors was evaluated via intra-rater reliability analysis using Mcdonald’s omega (Ω) coefficient and intraclass correlation coefficient (ICC), with values of at least 0.75 indicating satisfactory composite reliability. The minimum detectable change of the SSQOL-12 factor was calculated as the standard error of the measurement multiplied by 1.96 at a 95% confidence level. Test–retest reliability was examined via the bivariate correlation of the SSQOL-factors across Time 0 and Time 1. Examination of test–retest reliability typically adopts a target time of 2 weeks as the most commonly recommended interval^48^. Given the focus of SSQOL-12 on quality of life and the chronic recovery process of stroke, the present study adopted a longer time interval of 2 months as a reasonable time for clinical changes to occur in stroke survivors.

The second step evaluated the measurement invariance of the SSQOL-12 across 2 months. Multiple-group CFA estimated the configural invariance model with different factor loadings and item thresholds across time. Metric and scalar invariance models were then estimated to constrain the factor loadings and item thresholds to be equal across time, respectively^31^. Model identification was achieved by fixing the factor variances at one and autoregressive residual correlations were specified for each SSQOL-12 item between the two time points. Model comparison was evaluated via the chi-square difference test and model fit indices^49^. A change of ≥ − 0.01 for ΔCFI and a change of ≤ 0.015 for ΔRMSEA constituted support for scalar measurement invariance ^50^. Structural invariance tests examined the equality of factor means across time on a standardized metric. Apart from the dropout from Time 0 to Time 1, missing data were minimal (≤ 3%) in the present study. Missing data were handled under the missing-at-random assumption^51^ and attrition analysis examined the missing completely at random assumption of the study dropouts.

Thirdly, the concurrent, convergent, and divergent validity of the SSQOL-12 was evaluated via correlation analyses. Concurrent validity was evaluated with reference to the generic quality of life as measured by SF-12; convergent validity was evaluated with reference to hope and self-esteem; and divergent validity was evaluated with reference to the degree of disability, anxiety, and depression. Apart from bivariate correlation analysis, we conducted partial correlation analysis after controlling for demographic characteristics (gender and age) and clinical characteristics (type of stroke and onset time of stroke event). To evaluate the incremental utility of SSQOL-12 over the SF-12, we examined the partial correlations among SSQOL-12 and the convergent/divergent measures after controlling for SF-12. Statistical significance was set at 0.05 in the present study. The dataset analyzed in the present study was available in the form of a supplementary file (Supplementary data 1).

## Results

### Sample profile and attrition analysis

Table 1 presents the profiles of the respondents at baseline. The participants were middle-aged with an interquartile age range from 50 to 61. More than half were male, married, attained secondary education, and experienced ischemic stroke. The onset age of stroke was 51.4 years (SD = 8.4) with an interquartile range of 0.85–5.90 years for stroke duration. Half (51%) of the sample reported slight disability and a quarter (26%) reported moderate disability. The sample showed average levels of hope and self-esteem and a lower level of functioning in the physical domain (M = 36.6, SD = 8.5) than the mental domain (M = 46.7, SD = 10.4). Attrition analysis did not reveal significant differences between the study completers (N = 148) and dropouts (N = 36) in most of the study variables (*p* = 0.06–0.89) except for a significantly lower degree of disability (Cohen *d* = 0.16, *p* = 0.028) for the dropouts (mRS score = 1.86) than the study completers (mRS score = 2.18).

Table 2 displays the distribution of the scores for the 12 SSQOL items. Ceiling effects were found for 4 of the 12 items (language, mobility, self-care, and vision). The respondents reported slightly lower levels of functioning in areas such as interference with personal life, social life, upper extremity, and housework.

**Table 2:** Distribution of the scores of the 12 SSQOL items at baseline.

SSQOL-12 item	Mean (SD)	Total help/Strongly agree	A lot of help/Moderately agree	Some help/Neither agree nor disagree	A little help/Moderately disagree	No help needed/Strongly disagree
		N (%)	N (%)	N (%)	N (%)	N (%)
1. Energy	3.35 (1.26)	18 (10)	30 (16)	47 (26)	48 (26)	41 (21)
2. Family roles	2.93 (1.25)	28 (15)	42 (23)	51 (28)	40 (22)	23 (13)
3. Language	3.62 (1.28)	13 (7)	26 (14)	40 (22)	42 (23)	**62 (34)**
4. Mobility	3.45 (1.25)	15 (8)	29 (16)	43 (23)	52 (28)	**45 (25)**
5. Mood	3.35 (1.24)	18 (10)	28 (15)	48 (26)	52 (28)	38 (21)
6. Personality	3.38 (1.17)	8 (4)	37 (20)	58 (32)	39 (21)	42 (23)
7. Self-care	3.39 (1.44)	24 (13)	34 (18)	35 (19)	29 (16)	**62 (34)**
8. Social roles	2.84 (1.36)	38 (21)	44 (24)	40 (22)	33 (18)	29 (16)
9. Thinking	3.21 (1.26)	17 (9)	43 (23)	43 (23)	46 (25)	35 (19)
10. Upper extremity	3.01 (1.30)	24 (13)	51 (28)	38 (21)	41 (22)	30 (16)
11. Vision	3.80 (1.23)	9 (5)	25 (14)	31 (17)	47 (26)	**72 (39)**
12. Work	3.11 (1.31)	26 (14)	35 (19)	50 (27)	38 (21)	35 (19)

N = 184. The ceiling effects for four SSQOL items are bolded. SSQOL = Stroke-Specific Quality of Life Scale.

### Construct validity

**Figure 1:**
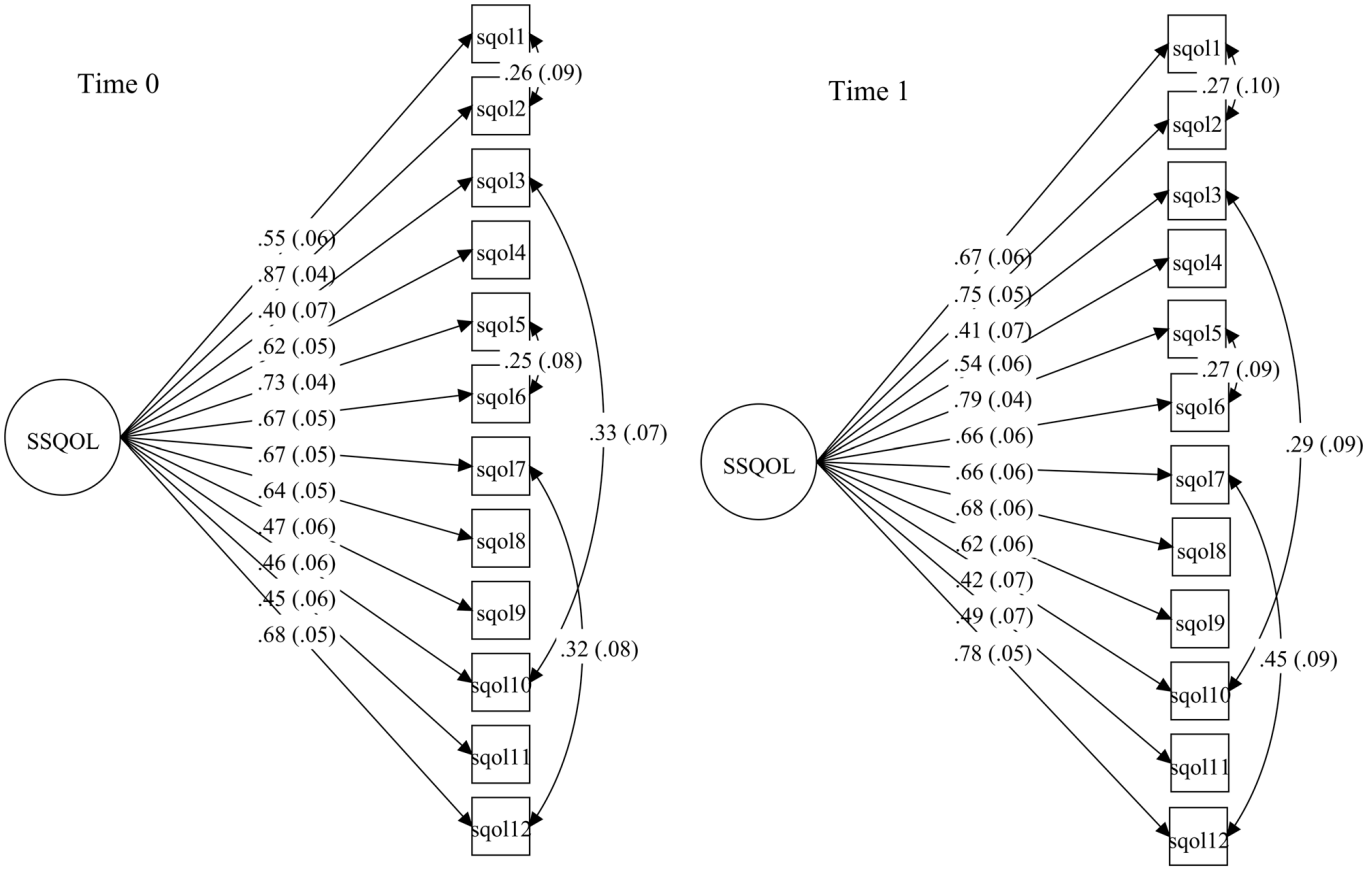
Factor structure of the modified 1-factor model of the 12-item Stroke-Specific Quality of Life Scale at baseline (Time 0) and 2-month follow-up (Time 1).

At Time 0 and Time 1, the 2-factor CFA model provided a better model fit than the 1-factor model (Δχ^2^ = 8.90–13.0, Δ*df* = 1, *p* < 0.01). From Table 3, neither model provided an adequate fit to the data with CFI and TLI < 0.95 and RMSEA > 0.10. Examination of modification indices suggested four residual correlations between item 1 (“fatigue”) and item 2 (“interference with personal life”), item 3 (“language”) and item 10 (“upper extremity”), item 5 (“mood”) and item 6 (“personality”), and item 7 (“self-care”) and item 12 (“housework”). Addition of these residual correlations led to an acceptable approximate fit (CFI and TLI ≥ 0.95, RMSEA ~ 0.08, and SRMR < 0.06) for the modified 1-factor model. The modified 2-factor model did not provide a superior fit over the modified 1-factor model (Δχ^2^ = 0.12–2.24, Δ*df* = 1, *p* = 0.13–0.73). The two factors in the 2-factor model were extremely highly correlated (*r* = 0.95–0.99). The potential factor redundancy supported a unidimensional factor structure for the SSQOL-12. Figure 1 depicts the factor structure of the modified 1-factor model at both Time 0 and Time 1. All of the factor loadings were substantial (λ = 0.40–0.87) at Time 0 and Time 1. The four residual correlations among the SSQOL-12 items were significant and moderate (*r* = 0.25–0.45, *p* < 0.05).

**Table 3:** Fit indices of confirmatory factor analysis models for the Stroke-Specific Quality of Life Scale at baseline and 2-month follow-up.

Model	Specification	χ^2^	df	RMSEA	CFI	TLI	SRMR	Δχ^2^ test (Δ df)	
Baseline (N = 184):							Model		
1	1-factor	171.7*	54	0.109	0.927	0.911	0.062		
2	2-factor	160.1*	53	0.105	0.934	0.918	0.060	1 versus 2	13.0* (1)
3	Modified 1-factor	117.0*	50	0.085	0.959	0.946	0.053		
4	Modified 2-factor	115.2*	49	0.086	0.959	0.945	0.052	3 versus 4	2.24 (1)
2-month follow-up (N = 148):									
5	1-factor	150.1*	54	0.110	0.940	0.927	0.060		
6	2-factor	143.9*	53	0.108	0.943	0.929	0.060	5 versus 6	8.90* (1)
7	Modified 1-factor	95.7*	50	0.079	0.971	0.962	0.048		
8	Modified 2-factor	95.5*	49	0.080	0.971	0.961	0.048	7 versus 8	0.12 (1)

*p < 0.01; df = degree of freedom; RMSEA = root mean square error of approximation; CFI = comparative fit index; TLI = Tucker-Lewis index; SRMR = standardized root mean square residuals; Δχ^2^ test = chi-square difference test.

### Reliability

In terms of internal consistency, the SSQOL-12 factor showed good levels of Mcdonald’s omega (Ω = 0.853, 95% CI = 0.821–0.885) and intraclass correlation coefficient (ICC = 0.858, 95% CI = 0.826–0.887) at Time 0. Good levels of internal consistency were found at Time 1 (Ω = 0.862, 95% CI = 0.829–0.896; ICC = 0.864, 95% CI = 0.829–0.894). For the SSQOL-12 factor, the standard error of measurement was 3.68 and the minimum detectable change was found to be 7.22 at a 95% confidence level. The SSQOL-12 factor at Time 0 was positively and strongly correlated (*r* = 0.70, *p* < 0.01) with the SSQOL-12 factor at Time 1.

### Measurement invariance across time

Table 4 shows the fit indices of measurement invariance models of the SSQOL-12 across time. The configural invariance model provided an adequate fit (CFI ≥ 0.95, RMSEA = 0.06, and SRMR = 0.065) to the data. The metric invariance model provided better model fit indices over the configural invariance model without significant difference (Δχ^2^ = 19.4, Δ*df* = 12, *p* = 0.08) in chi-square difference test. The scalar invariance model, which fixed the four thresholds of the 12 items to be equal across time, showed improved RMSEA and TLI over the metric model without significant difference (Δχ^2^ = 52.5, Δ*df* = 47, *p* = 0.27) in chi-square difference test. These findings supported scalar measurement invariance across time. The latent mean of the SSQOL-12 factor showed a non-significant increase from Time 0 to Time 1 (standardized mean difference = 0.16, *SE* = 0.08, *p* = 0.051). The model with invariant residual covariances did not display a significantly worse fit (Δχ^2^ = 4.44, Δ*df* = 4, *p* = 0.35) than the scalar model.

**Table 4:** Measurement invariance tests of the modified 1-factor model for the Stroke-Specific Quality of Life Scale across time (N = 184).

Invariance model	χ^2^	df	RMSEA	CFI	TLI	SRMR	Δχ^2^	Δdf	*p*
Configural	385.1*	231	0.060	0.951	0.942	0.065			
Metric	387.2*	243	0.057	0.954	0.948	0.070	19.4	12	0.08
Scalar	436.9*	290	0.052	0.953	0.956	0.071	52.5	47	0.27
Residual covariance	440.2*	294	0.052	0.954	0.956	0.071	4.44	4	0.35

*p < 0.01; χ^2^ = chi-square; df = degree of freedom; RMSEA = root mean square error of approximation; CFI = comparative fit index; TLI = Tucker-Lewis index; SRMR = standardized root mean square residuals; Δχ^2^ = chi-square difference.

### Concurrent, convergent, and divergent validity

Table 5 presents the correlations and partial correlations between the SSQOL-12 factor and demographic covariates, SF-12, coping variables, and distress variables at baseline. Bivariate correlation analysis found higher levels of SSQOL for male survivors (*r* = 0.17, *p* < 0.05) and survivors of ischemic stroke (*r* = 0.24, *p* < 0.01). Age and onset time of stroke were not significantly associated (*p* = 0.14–0.55) with the SSQOL factor. SSQOL was positively and moderately to strongly associated with hope and self-esteem (*r* = 0.41–0.52, *p* < 0.01) and SF-12 (*r* = 0.60, *p* < 0.01). SSQOL was negatively and moderately to strongly associated with the degree of disability, anxiety, and depression (*r* = − 0.38 to − 0.61, *p* < 0.01). Controlling for demographic and clinical characteristics, the partial correlational analysis found largely equivalent correlations between the SSQOL and other study variables. Controlling for SF-12, the association between SSQOL and anxiety became non-significant (*r* = − 0.04, *p* = 0.60). SSQOL remained significantly and positively linked with hope and self-esteem (*r* = 0.26–0.27, *p* < 0.01) and negatively linked with the degree of disability and depression (*r* = − 0.34 to − 0.40, *p* < 0.01).

**Table 5:** Correlations and partial correlations between the SSQOL-12 factor and demographic covariates, SF-12, coping variables, and distress variables at baseline.

	SSQOL-12 factor		
	No control variables	Control variables: Male, age, stroke type, onset time of stroke	Control variables: Male, age, stroke type, onset time of stroke, SF-12
Variables	*r*	*r*	*r*
Male	0.17*	–	–
Age	0.11	–	–
Ischemic stroke	0.24**	–	–
Onset time of stroke	− 0.05	–	–
SF-12	0.60**	0.57**	–
Hope	0.41**	0.46**	0.26**
Self-esteem	0.52**	0.53**	0.27**
Modified Rankin scale	− 0.43**	− 0.42**	− 0.34**
Anxiety	− 0.38**	− 0.37**	− 0.04
Depression	− 0.61**	− 0.63**	− 0.40**

N = 178–183; *p < .05; **p < .01; SSQOL = stroke-specific quality of life; SF-12 = Short Form Health Survey.

## Discussion

The present study conducted a systematic evaluation of the psychometric properties of the SSQOL-12 in a sample of Chinese stroke survivors in Hong Kong. In terms of construct validity, the extreme inter-factor correlations (*r* = 0.95–0.99) imply factor redundancy and do not support two factors in the present sample. Modification indices suggested four residual correlations to be the source of model misfit in the 1-factor CFA model at Time 0. The same modified 1-factor structure was replicated in the follow-up data. The rejection of χ^2^ test of exact fit for all CFA models could be attributed to inflated Type I errors of χ^2^ test for categorical models with small sample size (N < 200) in a methodological study^52^. The present results support the use of the total score as a parsimonious composite score on the stroke-specific quality of life.

From a theoretical point of view, the first residual correlation between item 1 (“fatigue”) and item 2 (“interference with personal life”) matches with the proximity between fatigue and interference in daily functioning among patients with chronic diseases such as cancer^53^ and inflammatory bowel disease^54^. Feelings of tiredness were not uncommon and could interfere with the functioning in daily lives of stroke survivors^55,56^. The second residual correlation between item 3 (“language”) and item 10 (“upper extremity”) could be explained by the shared neural substrates in the motor control system for spoken languages and arm gestures in Broca’s area^57^. A previous study^58^ found co-occurrence of language changes with motor changes following task specific training in stroke survivors. The third residual correlation could be attributed to the similar wordings (“lack of”) for item 5 (“lack of self-confidence”) and item 6 (“lack of patience towards others”). Both items tapped into psychological feelings of inferiority of the stroke survivors. For the fourth residual correlation, since item 7 focused on self-care in terms of the preparation of food, this item shared some overlaps with item 12 (“housework”) as both items referred to some forms of housework.

The SSQOL factor showed good levels of internal consistency in terms of omega and ICC at Time 0 and Time 1. The longitudinal measurement invariance tests supported the equality of factor loadings, item thresholds, and residual correlations for the SSQOL-12 items. These results lend support to unbiased and meaningful comparisons of temporal changes in the SSQOL-12 score over 2 months. The SSQOL-12 could act as a valid outcome measure of the stroke-specific quality of life for rehabilitation programs that usually last several months. An increase of at least 7.22 in the SSQOL total score would be necessary to attribute the improvement in stroke-specific quality of life to an intervention and not to the individual’s growth. The present study only examined the measurement invariance of the SSQOL-12 over a shorter timeframe of 2 months. Given the chronic recovery process, future research should investigate the longitudinal invariance of the SSQOL-12 over a longer timeframe of several years^59^.

In the present study, the gender difference in SSQOL is in line with previous findings on worse functional recovery and quality of life in female stroke survivors^60^. Consistent with previous findings^61,62^, patients with ischemic stroke showed higher levels of SSQOL than those with haemorrhagic stroke. The moderate to strong correlation between the SSQOL-12 and SF-12 scores support the concurrent validity of the SSQOL-12 in associating with generic QoL. Correlations in the expected directions and magnitudes were found for the SSQOL-12 factor with the convergent (hope and self-esteem) and divergent (degree of disability, anxiety, and depression) measures. These results offer empirical support to the convergent and divergent validity of the SSQOL-12. After controlling for SF-12, most of the associations between the SSQOL and the convergent/divergent measures (except for anxiety) remained statistically significant. These results imply incremental utility for using the SSQOL-12 over SF-12 to capture stroke-specific domains of quality of life of the survivors.

The unidimensional structure aligns with recent literature^63^ on the importance of mind–body connections among stroke survivors. From a clinical perspective, the present sample displayed an average total score of 39.5 for the SSQOL-12, which was lower than the level (Mean = 43.4) reported in a previous study^28^. The present sample also showed a lower level of generic QoL than previous studies^40,41^. Though stroke in young adults comprised 10%-15% of all stroke cases, previous studies^64–66^ have reported an increased incidence of stroke in young adults. The long-term prognosis could be challenging for the younger stroke survivors in terms of cardiovascular disease risks, psychosocial problems, and prevention of secondary stroke^67,68^. Modifiable risk factors such as smoking, hypertension, and dyslipidemia are prevalent in younger stroke survivors^69^. In Hong Kong, standard physiotherapy is the mainstream rehabilitation treatment for stroke survivors. There is a potential service gap in providing tailored rehabilitation programs to address the unmet needs of these stroke survivors in psycho-emotional, nutritional, social, and environmental domains^70,71^. Randomized controlled trials should evaluate the effects of patient-centered self-management interventions on their functional rehabilitation and mind–body interventions in improving their lifestyle and quality of life.

### Study limitations

There are several limitations in this study. First, the participants were recruited via convenience sampling and there could likely be self-selection biases. The selective attrition of those with a lower degree of disability could imply potential response and attrition biases. The present findings might not be generalized to other samples such as older stroke survivors. Further studies could examine the measurement invariance of the SSQOL-12 across gender, age groups and cultural contexts. Second, the concurrent, convergent and divergent validity of the SSQOL-12 was examined only at Time 0. The cross-sectional design could not differentiate the directions of relationships between SSQOL and emotional functioning and coping. Further studies could evaluate the concurrent validity of the SSQOL-12 with other stroke outcome measures such as Young Stroke Questionnaire^72^ in younger stroke survivors.

Third, all of the measures of quality of life and mental well-being were self-reported by the stroke survivors. The common method variance may introduce bias to the parameter estimates. Alternative forms of assessments such as functional assessments and observer ratings are recommended. Further studies could investigate the predictive role of dysphagia severity^73^ and life-space assessment^74^ in relation to SSQOL among stroke survivors. Fourth, the number of estimated parameters in the CFA ranged from 60 to 64 and the relatively low ratios of sample size to the number of parameters could bias the reliability of parameter estimates. Moshagen and Musch^75^ suggested that a sample size of around N = 200 could produce robust factor loadings and latent covariance for the WLSMV estimator. Although the four residual correlations were successfully replicated at Time 1, they could arise as a result of the idiosyncratic sample characteristics.

### Conclusion

The present study contributed to a better understanding of the psychometric properties of the SSQOL-12 in a sample of stroke survivors in Hong Kong. Empirical support was found for a robust 1-factor structure with adequate construct validity, reliability, convergent validity, and scalar measurement invariance across 2 months. It is recommended to conceptualize the SSQOL-12 as a unidimensional measure of the stroke-specific quality of life among stroke survivors in the Chinese context.

## Supplementary Information

Supplementary Information.

## Data Availability

All data generated or analysed during this study are included as the Supplementary Data file in this published article.
